# Whole-Genome Sequencing of *Sinocyclocheilus maitianheensis* Reveals Phylogenetic Evolution and Immunological Variances in Various *Sinocyclocheilus* Fishes

**DOI:** 10.3389/fgene.2021.736500

**Published:** 2021-10-05

**Authors:** Ruihan Li, Xiaoai Wang, Chao Bian, Zijian Gao, Yuanwei Zhang, Wansheng Jiang, Mo Wang, Xinxin You, Le Cheng, Xiaofu Pan, Junxing Yang, Qiong Shi

**Affiliations:** ^1^ College of Life Sciences, University of Chinese Academy of Sciences, Beijing, China; ^2^ Shenzhen Key Lab of Marine Genomics, Guangdong Provincial Key Lab of Molecular Breeding in Marine Economic Animals, BGI Academy of Marine Sciences, BGI Marine, BGI, Shenzhen, China; ^3^ State Key Laboratory of Genetic Resources and Evolution, The Innovative Academy of Seed Design, Yunnan Key Laboratory of Plateau Fish Breeding, Kunming Institute of Zoology, Chinese Academy of Sciences, Kunming, China; ^4^ Hunan Engineering Laboratory for Chinese Giant Salamander’s Resource Protection and Comprehensive Utilization, and Key Laboratory of Hunan Forest and Chemical Industry Engineering, Jishou University, Zhangjiajie, China; ^5^ Key Laboratory for Conserving Wildlife with Small Populations in Yunnan, Faculty of Biodiversity Conservation, Southwest Forestry University, Kunming, China; ^6^ BGI-Yunnan, Kunming, China

**Keywords:** *Sinocyclocheilus maitianheensis*, whole genome sequencing, assembly, annotation, phylogeny, immunity, cave adaptation

## Abstract

An adult *Sinocyclocheilus maitianheensis*, a surface-dwelling golden-line barbel fish, was collected from Maitian river (Kunming City, Yunnan Province, China) for whole-genome sequencing, assembly, and annotation. We obtained a genome assembly of 1.7 Gb with a scaffold N50 of 1.4 Mb and a contig N50 of 24.7 kb. A total of 39,977 protein-coding genes were annotated. Based on a comparative phylogenetic analysis of five *Sinocyclocheilus* species and other five representative vertebrates with published genome sequences, we found that *S. maitianheensis* is close to *Sinocyclocheilus anophthalmus* (a cave-restricted species with similar locality). Moreover, the assembled genomes of *S. maitianheensis* and other four *Sinocyclocheilus* counterparts were used for a fourfold degenerative third-codon transversion (4dTv) analysis. The recent whole-genome duplication (WGD) event was therefore estimated to occur about 18.1 million years ago. Our results also revealed a decreased tendency of copy number in many important genes related to immunity and apoptosis in cave-restricted *Sinocyclocheilus* species. In summary, we report the first genome assembly of *S. maitianheensis*, which provides a valuable genetic resource for comparative studies on cavefish biology, species protection, and practical aquaculture of this potentially economical fish.

## Introduction

Cave-restricted animals live in dark subterranean environments. They have evolved over time to adapt to the cave environments through various trait changes in morphology, behavior, and physiology (Jeffery, 2001). Cavefishes have degraded eyes, less body pigments, lower immune activities, and decrease in circadian rhythms in comparison to surface-dwelling fishes (Jeffery, 2009; Qiu et al., 2016; Yang et al., 2016; Krishnan and Rohner, 2017). As a compensation, the nonvisual sensory system of cavefishes is usually enhanced, such as development of extra taste buds and increased vibration attraction behavior (Yoshizawa et al., 2010; Yang et al., 2016). These facts of cavefishes are extremely interesting and worth to be explored with more investigations.

Previously, we have proved that cavefishes have fewer copies of major histocompatibility complex–related gene families (genes of cell surface proteins essential for acquired immune system) than surface-dwelling and semi–cave-dwelling counterparts, possibly suggesting relatively lower immune activities in cavefishes (Qiu et al., 2016), which may be a specific strategy for cave adaptation. Cave-restricted Mexican tetra (*Astyanax mexicanus*) also shows a big increase in appetite, but its fatty liver did not affect this fish’s health (Aspiras et al., 2015), implying that there may be some other immune-related molecular mechanisms for fighting inflammation in cavefishes (Xiong et al., 2019). A recent study (Peuß et al., 2020) proposed that organisms in various environments have developed differential immune strategies with innate immune degradation and T-cell overexpression in cavefishes. However, many of these putative molecular mechanisms are not fully understood.

*S. maitianheensis* lives originally in the surface of Maitian river in Kunming City, Yunnan Province, China (Supplementary Figure S1). Some *Sinocyclocheilus* fishes are also residents in caves*. S. maitianheensis* can therefore be used as a control for comparative studies on cave adaptation. Meanwhile, as a genus of state second-class protection in China, *Sinocyclocheilus* has been propagated with a series of artificial breeding for protection from extinction (Yin et al., 2021). Various *Sinocyclocheilus* species likely shared tetraploid origin and have 96 chromosomes that are twice of most teleosts (Heng et al., 2002). The genome diversity makes fishes in this genus as good models for studying cave adaptation and phylogenetic evolution. Although there are some reports on morphological and mitochondrial genomic evolution of various *Sinocyclocheilus* species (Wu et al., 2010; He et al., 2012; Chen Y.-Y. et al., 2018), whole genome–based comparative studies are rare, except for three representative *Sinocyclocheilus* fishes that we published before (Yang et al., 2016).

Here, we performed whole-genome sequencing, assembly, and annotation of *S. maitianheensis* and subsequently conducted comparative genomic analysis and immune-gene inquiry with four other *Sinocyclocheilus* counterparts (including surface-dwelling *Sinocyclocheilus grahami,* semi–cave-dwelling *Sinocyclocheilus rhinocerous*, and cave-restricted *Sinocyclocheilus anophthalmus* and *Sinocyclocheilus anshuiensis*; their genome sequences are publicly available). Our main purpose is to provide a genetic resource for in-depth studies on cave adaption and cavefish biology. Our study can also contribute to the species protection and exploitation of potentially economical value for *S. maitianheensis*.

## Materials and Methods

### Sampling, Library Constructing, and Genome Sequencing

An adult *S. maitianheensis* was collected from Maitian river in Kunming City, Yunnan Province, China, for genome and transcriptome sequencing. Genomic DNAs were extracted from muscle sample. Seven Illumina paired-end sequencing libraries (with insert sizes of 270 bp, 500 bp, 800 bp, 2 kb, 5 kb, 10 kb, and 20 kb, respectively) were constructed for a routine shotgun whole-genome sequencing in an Illumina HiSeq 2,500 platform (San Diego, CA, United States). SOAPfilter v2.2 (Li R. et al., 2009) (-z -p -g 1 -f -o clean -M 2 -f 0) was used to filter reads. Duplicate reads from polymerase chain reactions, those reads with 10 or more nonsequencing bases (Ns), adapter sequences, and bases with low quality were removed.

Total RNAs were extracted from muscle, skin, eye, liver, heart, and brain for construction of individual cDNA library. cDNAs were then sequenced in an Illumina HiSeq X platform and filtered by SOAPnuke v1.0 (Chen Y. et al., 2018) with optimized parameters [-l 10 (default: 5) -q 0.2 (default: 0.5) -n 0.05 -c 0 -Q 2 (default: 1)]. Reads with nonsequenced (N) base ratio of more than 5% or low-quality base (base quality ≤10) ratio of greater than 20% were discarded to generate a new set of higher-quality reads for subsequent transcriptome-based annotation.

### Genome Survey, *de Novo* Assembly, and Assessment

Genome size was estimated via the routine 17-mer frequency distribution analysis with the following formula: genome size = *K*_num_/*K*_depth_, where *k*_num_ is the number of k-mers obtained from reads, and *K*_depth_ is the expected depth of k-mer at a maximum frequency (Song et al., 2016). Two Illumina short-insert libraries (500 and 800 bp) were used for this 17-mer analysis.

The genome assembly strategy includes three steps. First, SOAPdenovo2 v2.04.4 (Luo et al., 2012) was applied to produce primary and final scaffolds with the following parameters: pregraph -K 27 -p 16 -d 1; contig -M 1; scaff -F -b 1.5 -p 16. Contigs and primary scaffolds were generated by using filtered reads from short-insert libraries (200, 500, and 800 bp), and the final scaffolds were constructed by mapping long-insert libraries (2, 5, 10, and 20 kb) onto the primary scaffolds. Second, gaps in scaffolds were then filled in two rounds using paired-end reads from the three short-insert libraries (270, 500, and 800 bp) via GapCloser v1.12 and v1.10 (Li R. et al., 2009) (-t 8 -l 150 and -t 25 -p 25, respectively). Finally, SSPACE V2.0 (Boetzer and Pirovano, 2014) (-k 5 -T 25 -g 2) was used to further extend and fill up both contigs and scaffolds. Completeness assessment of the final genome assembly was performed by BUSCO v5.2.2 (Simão et al., 2015; Manni et al., 2021) (e-value ≤1e-3) with the popular actinopterygii_odb10 database.

### Repeat Annotation

Repeat sequence annotation is composed of three routine methods, including *de novo* annotation, homology prediction, and tandem repeat prediction. First, we used RepeatModeller v1.04 (Chen, 2004) and LTR-FINDER v1.0.6 (Xu and Wang, 2007) to construct a local *de novo* repeat reference, and then our assembled genome was aligned to this reference library by RepeatMasker v4.06 (Chen, 2004). In addition, RepeatMasker v4.06 (Chen, 2004) and RepeatProteinMask v4.06 (Chen, 2004) were applied for homology prediction after identification of transposable elements based on RepBase (Jurka et al., 2005). Moreover, Tandem Repeat Finder v4.09 (Benson, 1999) was separately used to predict comprehensive tandem repeats in our pipeline as previously reported (Liu et al., 2019; Zhao et al., 2021). Finally, these results from the aforementioned three methods are integrated by our in-house perl scripts (https://github.com/liruihanguo/Repeats_integration). These scripts separately classified each type of repeat, integrated all repeats, and then removed those overlaps to obtain a nonredundant repeat set.

### Gene Structure and Function Annotations

Two different methods were used for gene annotation to generate a total gene set, including homology annotation and transcriptome-based annotation (Bian et al., 2019). For the homology annotation, we downloaded protein sequences of four representative vertebrates from NCBI (Benson et al., 2006), including zebrafish (*Danio rerio*)*,* Japanese medaka (*Oryzias latipes*), and two *Sinocyclocheilus* fishes (*S. anshuiensis* and *S. rhinocerous*), to align them against our *S. maitianheensis* genome assembly by TBLASTn (e-value ≤1e-5) (Gertz et al., 2006). Each gene structure was predicted by GeneWise v2.4.2 (Birney et al., 2004). For the transcriptome-based annotation, Tophat v2.0.13 (Trapnell et al., 2009) was utilized to obtain potential genes by mapping transcriptome reads onto our assembled genome. Subsequently, Cufflink v2.2.1 (Trapnell et al., 2013) was applied to predict the structures of potential genes on the alignments sorted by samtools v1.1 (Li H. et al., 2009). Lastly, the final consensus gene set was integrated by MAKER v2.31.8 (Cantarel et al., 2008).

All these predicted genes were aligned onto several public databases, including Interpro (Hunter et al., 2009), KEGG (Kanehisa et al., 2017), TrEMBL (Boeckmann et al., 2003), and Swiss-Prot (Boeckmann et al., 2003), using BLASTp (McGinnis and Madden, 2004) (e-value ≤1e-5) to perform function annotation. These results were then assessed by comparing coding sequence (CDS) length, intron length, gene length, exon length, and exon number distributions with the four closely related *Sinocyclocheilus* species, including *S. anshuiensis* (Yang et al., 2016) (SAMN03320099. WGS_v1.1 in NCBI), *S. rhinocerous* (Yang et al., 2016) (SAMN03320098_v1.1), *S. grahami* (Yang et al., 2016) (SAMN03320097. WGS_v1.1), and *S. anophthalmus* [genome assembly was deposited at NCBI under accession no. PRJNA669129 (GCA_018155175.1)]. This unpublished genome of *S. anophthalmus* was sequenced by us on both Illumina Hiseq2500 and PacBio Sequel platforms using muscle genomic DNAs, and the final assembly of 1.9 Gb (with a contig N50 of 229.8 kb, a scaffold N50 of 309.9 kb, and prediction of 49,865 protein-coding genes) was assembled by combining the corrected long PacBio reads and the primary assembly from short Illumina reads by DBG2OLC v1.1 (Ye et al., 2016). We also assessed the completeness of these protein-coding gene sets by BUSCO v5.2.2 (Manni et al., 2021).

### Orthogroup Cluster

The protein sequences of *S. maitianheensis* and other ten representative species were used for clustering orthogroups and phylogenetic analyses. These vertebrates include four *Sinocyclocheilus* species (*S. anophthalmus*, *S. grahami*, *S. anshuiensis*, and *S. rhinocerous*), common carp (*Cyprinus carpio*, GCF_000951,615.1 in NCBI), zebrafish (GCF_000002035.6), Japanese medaka (GCF_002234675.1), and Asian arowana (*Scleropages formosus*, GCF_900964775.1)*,* as well as the outgroup of human (*Homo sapiens*, GCF_000001405.39) and mouse (*Mus musculus*, GCF_000001635.27)*.* We used BLASTp (McGinnis and Madden, 2004) (e-value ≤1e-5) to align these protein sequences with each other and OrthoMCL v2.0.92 (Fischer et al., 2011) with default parameters to identify orthologous genes and construct orthogroups.

### Phylogenetic and Divergence Time Analyses

Single-copy orthogroups were aligned using MUSCLE v3.8.31 (Edgar, 2004). Subsequently, conserved regions were obtained by Gblocks (Castresana, 2000), and the CDS regions of all single-copy genes from each species were connected to form a supergene for extraction of the 4d sites. We also constructed a phylogenetic tree by using PhyML v3.0 with the maximum likelihood method (Guindon et al., 2010). MCMCtree in the PAML package (Yang and Rannala, 2006) was used to estimate the divergence time of five *Sinocyclocheilus* fishes and other species by three calibration time points of fossil records (Benton and Donoghue, 2007), including 61.5–100.5 Mya for *H. sapiens* and *M. musculus*, 159.9–165.2 Mya for *D. rerio* and *O. latipes*, and 416.1–421.8 Mya for *D. rerio* and *H. sapiens*.

### 4dTv Analysis to Identify Specific Whole-Genome Duplication in *Sinocyclocheilus* Fishes

To estimate the *Sinocyclocheilus* specific whole-genome duplication **(**WGD) event, we performed a fourfold degenerative third-codon transversion (4dTv) analysis by comparing five *Sinocyclocheilus* genomes with zebrafish and common carp genome assemblies. The WGD periods were calculated by using the following formula [The recognized time of 3R WGD (∼320 Mya)/the 4dTv peak values of 3R WGD in *Sinocyclocheilus* (0.65–0.75)] * the 4dTv peak values of lineage-specific WGD of *Sinocyclocheilus* (0.04–0.05).

An all-to-all alignments of protein sequences from these seven genomes were applied by using BLASTp (McGinnis and Madden, 2004) with an e-value of 1e-5. Syntenic blocks between species were identified using i-ADHoRe 3.0 (Proost et al., 2012) with default parameters, and then homologous proteins were obtained. Subsequently, homologous pairs were aligned using MUSCLE (Edgar, 2004), after we retrieved these homologous protein sequences and converted them to nucleotide sequences. Lastly, we calculated and corrected the 4dTv values for each gene pair by using the HKY model in PAML package (Yang and Rannala, 2006).

### Identification of Immune Genes and Pseudogenes in P38 and Mitochondrial Pathways

Fifteen apoptosis-related genes were identified in five *Sinocyclocheilus* genomes and other five representative vertebrate genomes with high quality to investigate the gene copy number in P38 and mitochondrial pathway. These other five vertebrates include common carp, zebrafish, Japanese medaka, Asian arowana, and Mexican tetra (*A. mexicanus*, GCF_000372,685.1 in NCBI). Each genome sequence was used to construct a standard aligned database in the first place. Protein sequences of *tak1*, *tab1*, *ask1*, *fas*, *fasl*, *fadd*, *tnfa*, *cd40*, *cd40l*, *daxx*, *mkk4a*, *mkk4b*, *mkk6*, *bcl-2a*, and *bcl2l1* of zebrafish were downloaded from public uniport databases (Supplementary Table S1) as the queries. These protein sequences were then aligned onto above 10 genomes by TBLASTn (e-value ≤1e-5). The alignments with aligned ratio of less than 0.5, sequences similarity of less than 50%, and redundant data were filtered out to obtain the final hit alignments. Subsequently, target apoptosis-related genes were predicted by GeneWise v2.4.1 (Birney et al., 2004) from these 10 vertebrate genomes.

We performed a multiple-sequence alignment using the Muscle module in MEGA v7.0 (Kumar et al., 2016) to identify pseudogenes in each species indicated above. The whole open reading frames were performed with codon-based alignment to identify potential pseudogenes with irregular shifts of premature stop codon(s), codon frameshifts, or missing exon regions.

## Results and Discussion

### Summary of the Genome Assembly and Assessment

We generated a total of 236.7-Gb raw reads, among them approximately 179.5 Gb of clean reads were obtained after removal of low-quality data. For the transcriptome sequencing, a total of 39.3-Gb raw reads were generated. The genome size of *S. maitianheensis* was estimated to be about 1.8 Gb by the routine 17-mer frequency distribution analysis, because the *k*_num_ is 40,525,178,512, and the *K*_depth_ is 23 (see Supplementary Figure S2).

After *de novo* assembly and gap closing, the final genome assembly was 1.7 Gb in total length, with a scaffold N50 of 1.4 Mb, a contig N50 of 24.7 kb, and the GC content of 37.6% (Tables 1 and Supplementary Table S2). The final assembled genome accounted for 94.4% of the estimated genome size (1.8 Gb). For assessment of our genome assembly, we searched a total of 3,640 BUSCO (Benchmarking Universal Single-Copy Orthologs) groups and determined that 3,536 (97.1%) were complete (with 1,643 single-copy BUSCOs and 1,893 duplicated BUSCOs), suggesting a high completeness of our genome assembly for *S. maitianheensis.*

**TABLE 1:** Statistics of the genome assembly for *S. maitianheensis*.

Genome assembly	Parameter
Contig N50 (kb)	24.7
Contig number (>100 bp)	212,978
Scaffold N50 (Mb)	1.4
Scaffold number (>100 bp)	106,737
Total length (Gb)	1.7
Genome coverage (×)	139.2
The longest scaffold (Mb)	12.5
**Genome annotation**	**Parameter**
Protein-coding gene number	39,977
Mean transcript length (bp)	15,881
Mean exons per gene	8.6
Mean exon length (bp)	188.3
Mean intron length (bp)	1,728

We compared the genome assembly and annotation results for the five examined *Sinocyclocheilus* species and observed that their genome sizes are at a narrow range of 1.7–1.9 Gb (Supplementary Table S3). The largest genome is 1.9 Gb for *S. anophthalmus* (GCA_018155175.1 in NCBI) whose assembly was generated with both Illumina (next-generation) and PacBio (third-generation) sequencing reads, whereas others were sequenced only by an Illumina platform with relatively lower scaffold N50 values. GC contents of the five examined *Sinocyclocheilus* species ranged from 37.2 to 38.6% (Supplementary Table S3).

### Genome Annotation

For repeat annotation, we predicted 664,837,705-bp repeat elements (accounting for 39.4% of the genome assembly; Supplementary Table S4). Among them, 455.9 Mb of DNA repeat elements, 135.2 Mb of long terminal repeats (LTRs), 104.9 Mb of long interspersed nuclear elements (LINE), and 9.1 Mb of short interspersed nuclear elements (SINE) were detected (Supplementary Table S5). After homology and transcriptome-based annotations, our MAKER results integrated a total of 39,977 protein-coding genes with an average length of 15.9 kb (Supplementary Table S6).

For function annotation, 38,677 genes were aligned onto four public databases (Interpro, KEGG, TrEMBL, and Swiss-Prot) with function assignments, which account for 96.8% of the annotated genes (Supplementary Table S7). The distributions of CDS, intron, gene, and exon length in *S. maitianheensis* were generally similar to those in other four examined *Sinocyclocheilus* fishes (Supplementary Figure S3), which suggested a good reliability of the genome annotation for *S. maitianheensis.* However, there are still some distinctions among them, such as the higher percentages of CDS and genes at length approximately 1,000 bp in *S. maitianheensis*. Moreover, our annotation results reveal that *S. maitianheensis* has less protein-coding gene number (39,977) than the other four *Sinocyclocheilus* counterparts (49,865 for *S. anophthalmus*, 42,109 for *S. grahami*, 40,470 for *S. anshuiensis*, and 42,377 for *S. rhinocerous*)*.* The assessment of protein-coding genes among the five fishes showed that there were more missing BUSCOs in *S. maitianheensis* (Supplementary Table S8), which may lead to a lower amount of gene number. However, this divergence of gene number may be caused in part by different annotation methods*.*

### Summary of the Gene Orthogroups

**FIGURE 1:**
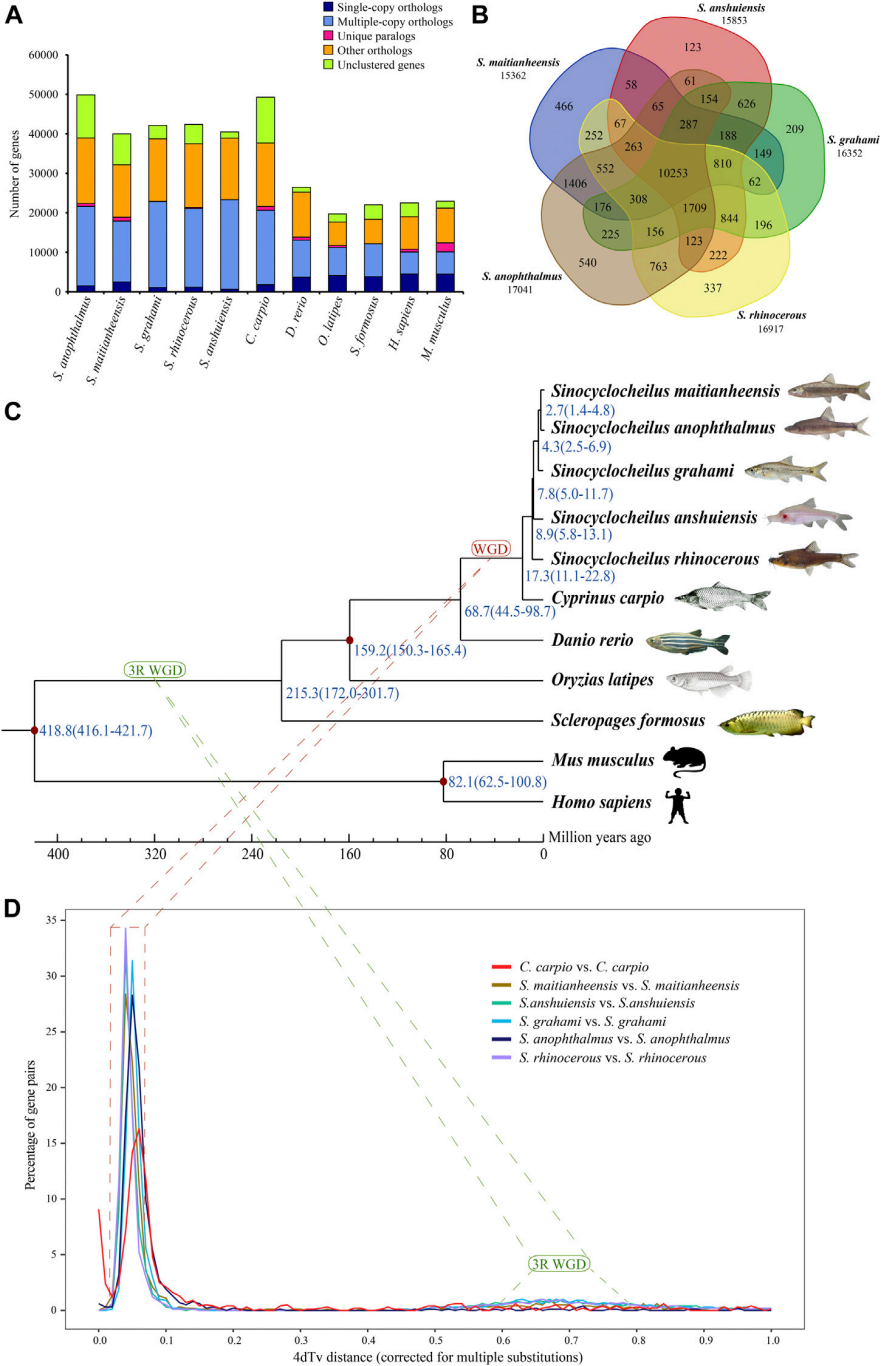
Orthogroups and an evolutionary analysis. **(A)** Distribution of homologous orthogroups among the examined 10 vertebrate species. **(B)** Orthogroup cluster of the five *Sinocyclocheilus* fishes, including *S. maitianheensis*, *S. anophthalmus*, *S. grahami*, *S. anshuiensis*, and *S. rhinocerous*. **(C)** A phylogenetic tree of various representative vertebrates including the five *Sinocyclocheilus* fishes. Diverge time is represented in blue, and the geographic time scale is in million years (for Mya). “WGD” represents the *Sinocyclocheilus*-specific WGD event (red), and “3R WGD” represents the third-round WGD event (green). **(D)** 4dTv distributions of self-alignments in five *Sinocyclocheilus* fishes, and common carp.

A total of 26,875 orthogroups were predicted for the five examined *Sinocyclocheilus* fishes and six other representative vertebrate species. For *S. maitianheensis*, 32,150 protein-coding genes were clustered into 15,617 orthogroups, although 7,827 genes were not clustered; the numbers of multiple-copy, single-copy orthologs, and unique paralogs were 15,400, 2,508 and 981, respectively (Figure 1A and Supplementary Table S9). We summarized the numbers of orthogroups shared with each other between the five *Sinocyclocheilus* in Figure 1B. There are 10,253 common orthogroups among these *Sinocyclocheilus* species*.*

### Phylogenetic Position of *S. maitianheensis*

A total of 191 common single-copy orthogroups among all the examined species were used for construction of a phylogenetic tree. A progressive evolutionary relationship in the five *Sinocyclocheilus* species revealed a new phylogeny based on both genome and transcriptome data (Figure 1C). This topology is consistent with the phylogenetic tree based on genomes (Yang et al., 2016) and mitochondrial genes (Zhang and Wang, 2018), but it is different from a previous report of a division into two branches, in which *S. maitianheensis*, *S. anophthalmus*, and *S. anophthalmus* are located in one branch, whereas *S. anshuiensis* and *S. rhinocerous* are located in another branch (Mao et al., 2021) based on the morphological trait of eyes. Therefore, our latest topology provides novel insights into detailed phylogeny for the five *Sinocyclocheilus* species at a genome level*.*

*S. maitianheensis* and *S. anophthalmus* diverged at approximately 2.7 (1.4–4.8) Mya, and the divergence time periods with *S. grahami, S. anshuiensis*, and *S. rhinocerous* were 4.3 (2.5–6.9), 7.8 (5.0–11.7), and 8.9 (5.8–13.1) Mya, respectively (Figure 1C). Surface-dwelling *S. maitianheensis* has the closest relationship with cave-restricted *S. anophthalmus*; however, there are huge differences in morphological traits between them. Interestingly, the geographical positions of both habitats are close, located in the same Yiliang County (Kunming City, Yunnan Province, China; Supplementary Figure S1). *S. anophthalmus* resides in a dark environment of several caves in Jiuxiang Town (Zhao and Zhang, 2009), whereas *S. maitianheensis* lives in the surface of Maitian river. Divergence time of the two *Sinocyclocheilus* species was relatively late, approximately 2.7 (1.4–4.8) Mya. Their separation may be due to a geographical isolation generated by the continuous uplift of the Yunnan–Guizhou Plateau after Himalayan orogeny (50–40 Mya) (Yin and Harrison, 2000), and some of their ancestors swam down along the Maitian river into these surrounding caves.

### Estimation of the Lineage-specific WGD in *Sinocyclocheilus*

Cyprinidae experienced a recent genome-wide duplication event (Larhammar and Risinger, 1994; David et al., 2003) after the third-round WGD (3R WGD, also known as teleost-specific WGD) (Jaillon et al., 2004). We performed a 4dTv analysis to estimate the timing of this recent lineage-specific WGD in *Sinocyclocheilus*. Self-alignments with paralogous gene pairs of *S. maitianheensis*, *S. anshuiensis*, *S. rhinocerous*, *S. anophthalmus*, *S. grahami*, and common carp displayed distinct peaks (Figure 1D). Their 4dTv distances were calculated to be 0.04, 0.04, 0.04, 0.05, 0.05, and 0.06, respectively. The peak values of the five *Sinocyclocheilus* fishes and common carp are very close (0.04–0.06), implying that these fishes might share the recent genome-wide duplication event.

In order to compare the recent specific WGD between *Sinocyclocheilus* fishes and common carp, we performed a 4dTv analysis on 13,579 orthologous gene pairs between *S. maitianheensis* and common carp genomes (Supplementary Figure S4). The peak values in each group of *C. carpio*–*C. carpio*, *S. maitianheensis*–*C. carpio*, and *S. maitianheensis*–*S. maitianheensis* were estimated to be 0.06, 0.04, and 0.04, respectively. The nearly overlapping of peaks indicated that *S. maitianheensis* and common carp might have undergone the recent specific WGD together. Based on the aforementioned 4dTv analyses and the construction of phylogenetic tree, we predicted that the carp-specific WGD occurred ∼18.1 Mya, before the evolutionary separation of *Sinocyclocheilus* fishes and common carp (∼17.3 Mya; Figure 1D). This estimate is little earlier before the specific WGD of common carp based on the divergence time of transposable elements (Xu et al., 2019).

The time estimation of the latest WGD in *Cyprinidae* is contentious, from ∼8.2 to ∼16 Mya (Larhammar and Risinger, 1994; David et al., 2003; Xu et al., 2014), although these studies almost focused on the common carp. However, a recent study of common carp defined a rough time range (9.7–23 Mya) and further predicted this time point to about 12.4 Mya (Xu et al., 2019). Our result of ∼18.4 Mya in the present study is within this time range, and the 4dTv analysis of *Sinocyclocheilus* fishes provides more evidence for the timing of the latest genome duplication in *Cyprinidae*.

### Copy Number Variations of Several Immune Genes in P38 and Mitochondrial Pathways

*S. anophthalmus* has evolved a series of traits to adapt to the caved environment. It had developed huge differences from its sister species (*S. maitianheensis*), such as loss of eyes and the semitransparent body (Zhao and Zhang, 2009). Therefore, compared to *S. maitianheensis*, *S. anophthalmus* is an independent cave species with many valuable traits that are waiting for in-depth explorations.

To investigate potential immunological variances in cave-restricted *Sinocyclocheilus* species, we identified the copy number of 15 immune genes within P38 and mitochondrial pathways in the five examined *Sinocyclocheilus* genomes (see detailed statistics in Supplementary Table S10). These genes include *tak1*, *tab1*, *ask1*, *fas*, *fasl*, *fadd*, *tnfa*, *cd40*, *cd40l, daxx*, *mkk4a, mkk4b*, *mkk6*, *bcl-2a*, and *bcl2l1* (Supplementary Figure S5). In general, five *Sinocyclocheilus* species and common carp usually own twofold copies compared to the other diploid teleosts. Interestingly, we found copies of some genes in the cave-restricted fishes. For *S. anophthalmus*, a copy of *ask1* (an apoptosis signal-regulating kinase) (Patel et al., 2019) was predicted as a pseudogene; only one copy of *bcl2l1* (encoding apoptotic regulators in BCL-2 family) (Warren et al., 2019) was retained in *S. anophthalmus* compared with *S. maitianheensis* that contained two copies; one more copy of *bcl-2a* and no copy of *mkk4a* were also observed in *S. anophthalmus* genome. In addition, among most of these genes we studied, another cave-restricted fish Mexican tetra (*A. mexicanus*) had fewer gene copy numbers than the other examined fishes. These variances in gene copy number imply that the apoptotic activity might have been decreased in cave-restricted fishes, which is consistent with our previous report of relatively lower immunity in cavefishes (Qiu et al., 2016). However, apoptotic activity is regulated by many factors, and more investigations should be done for in-depth verification.

In addition, we performed high-throughput identification of antimicrobial peptides (AMPs) (Yi et al., 2017; Mwangi et al., 2019). A total of 379, 551, 522, 545, and 552 putative AMP sequences were identified in *S. maitianheensis*, *S. rhinocerous*, *S. anshuiensis*, *S. anophthalmus*, and *S. grahami* genomes, respectively (Supplementary Table S11). Thrombin, histone, lectin, chemokine, scolopendin, and ubiquitin are the most abundant AMPs in the five examined *Sinocyclocheilus* species. The lowest number of AMP sequences in *S. maitianheensis* genome may be related to its least protein-coding genes in the five *Sinocyclocheilus* fishes.

## Conclusion

In summary, we reported the first genome assembly of *S. maitianheensis*, which provides a valuable genetic resource for comparative studies on cavefish biology, species protection and practical aquaculture of this potentially economical teleost fish. This genome assembly also supplies essential genomic data for in-depth genetic analysis. Based on these genomic data, we observed a close relationship between *S. maitianheensis* and *S. anophthalmus*. Some variations of gene copy number in the immune system might indicate the variation in immunity and apoptosis in cave-restricted *Sinocyclocheilus* species.

## Value of the Data

This is the first draft genome of a representative surface-dwelling Chinese golden-line barbel fish, *Sinocyclocheilus maitianheensis*. The final assembly was 1.7 Gb with a scaffold N50 of 1.4 Mb and a contig N50 of 24.7 kb.

The phylogenetic tree revealed that *S. maitianheensis* is close to *S. anophthalmus* (a cave-restricted species with similar locality). The divergence time between the two relatives is about 2.7 million years ago (Mya).

The 4dTv analysis demonstrated that the recent carp-specific WGD event occurred approximately 18.1 Mya.

A decrease in the copy number of many important immunological genes was observed in cave-restricted *Sinocyclocheilus* species.

## Data Availability

The datasets presented in this study can be found in online repositories. The names of the repository/repositories and accession number(s) can be found in the article/Supplementary Material.

## Abbreviations

*ask1*, apoptosis signal-regulating kinase 1, also known as mitogen-activated protein kinase 5 (*map3k5*); *bcl-2*, B-cell lymphoma 2; *bcl2l1*, *bcl-2*-like protein 1; *cd40*, tumor necrosis factor receptor superfamily member 5; *cd401*, tumor necrosis factor ligand superfamily member 5; *daxx*, death domain-associated protein 6; *fadd*, fas-associated protein with death domain; *fas*, tumor necrosis factor receptor superfamily member 6; *fasl*, tumor necrosis factor ligand superfamily member 6; *mkk4*, mitogen-activated protein kinase kinase 4, also named *map2k4*; *mkk6*, mitogen-activated protein kinase kinase 6, also named *map2k6*; *tab1*, mitogen-activated protein kinase 7-interacting protein 1; *tak1*, mitogen-activated protein kinase kinase kinase 7, also named *map3k7*; *tnfa*, Tumor necrosis factor ligand superfamily member 1, also named *tnf*.
