# Supplementary material for: Whole-Genome Sequencing of Sinocyclocheilus maitianheensis Reveals Phylogenetic Evolution and Immunological Variances in Various Sinocyclocheilus Fishes
Source: Front Genet. 2021 Oct 5;12:736500. doi: 10.3389/fgene.2021.736500 (PMC8523889; doi:10.3389/fgene.2021.736500)
Supplement: Supplementary file 1 [file DataSheet1.docx]

**Supplementary** **Figures and Tables**

**
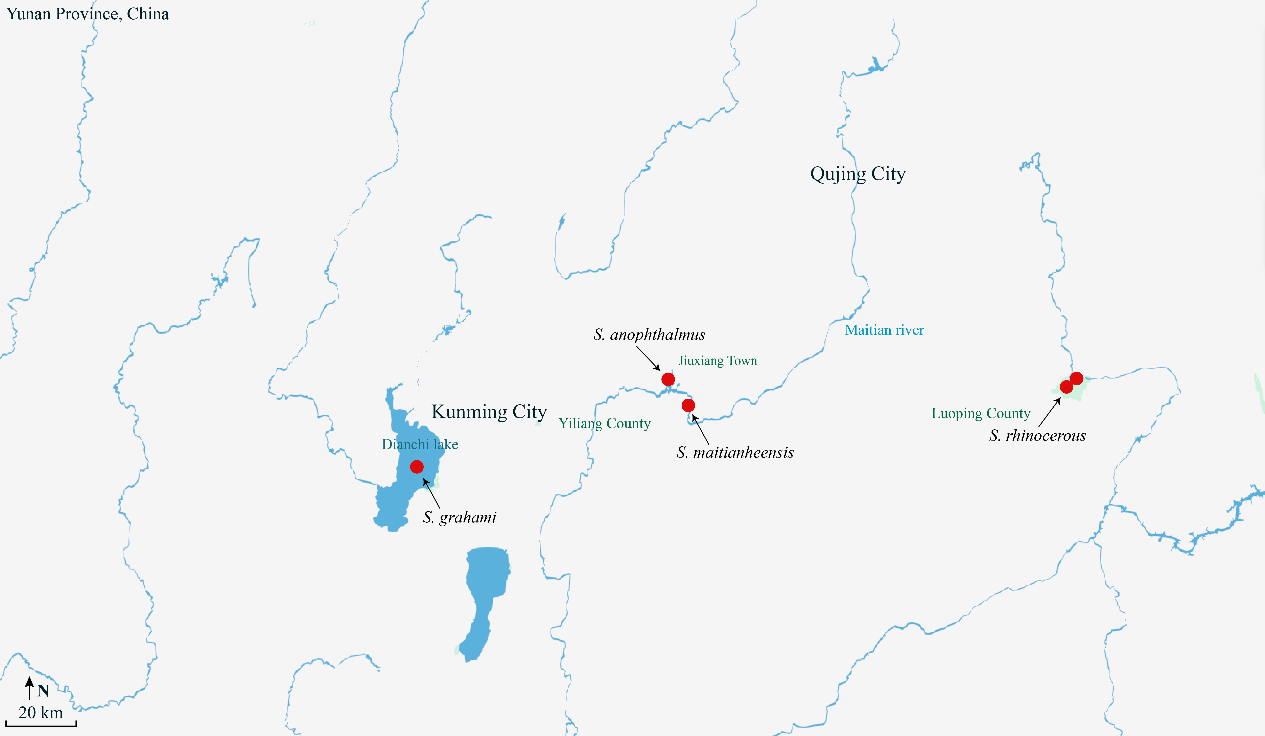
**

**Supplementary Figure 1** Geographical distribution of various *Sinocyclocheilus* fishes in Yunnan Province, China. Red points represent the distribution of each fish (Zhao and Zhang, 2009, *Environmental biology of fishes* **86**(1)**:** 163). *S. grahami* lives in Dianchi lake of Kunming City. *S. anophthalmus* and *S. maitianheensis* are both located in Yiliang County, Kunming City: *S. anophthalmus* lives in several caves of Jiuxiang Town, while *S. maitianheensis*lives in the surface of Maitian river. *S. rhinocerous* lives in surface and underground rivers, locating in Luoping County, Qujing City. This map was generated by editing a Baidu Map (http://lbsyun.baidu.com/products/map).


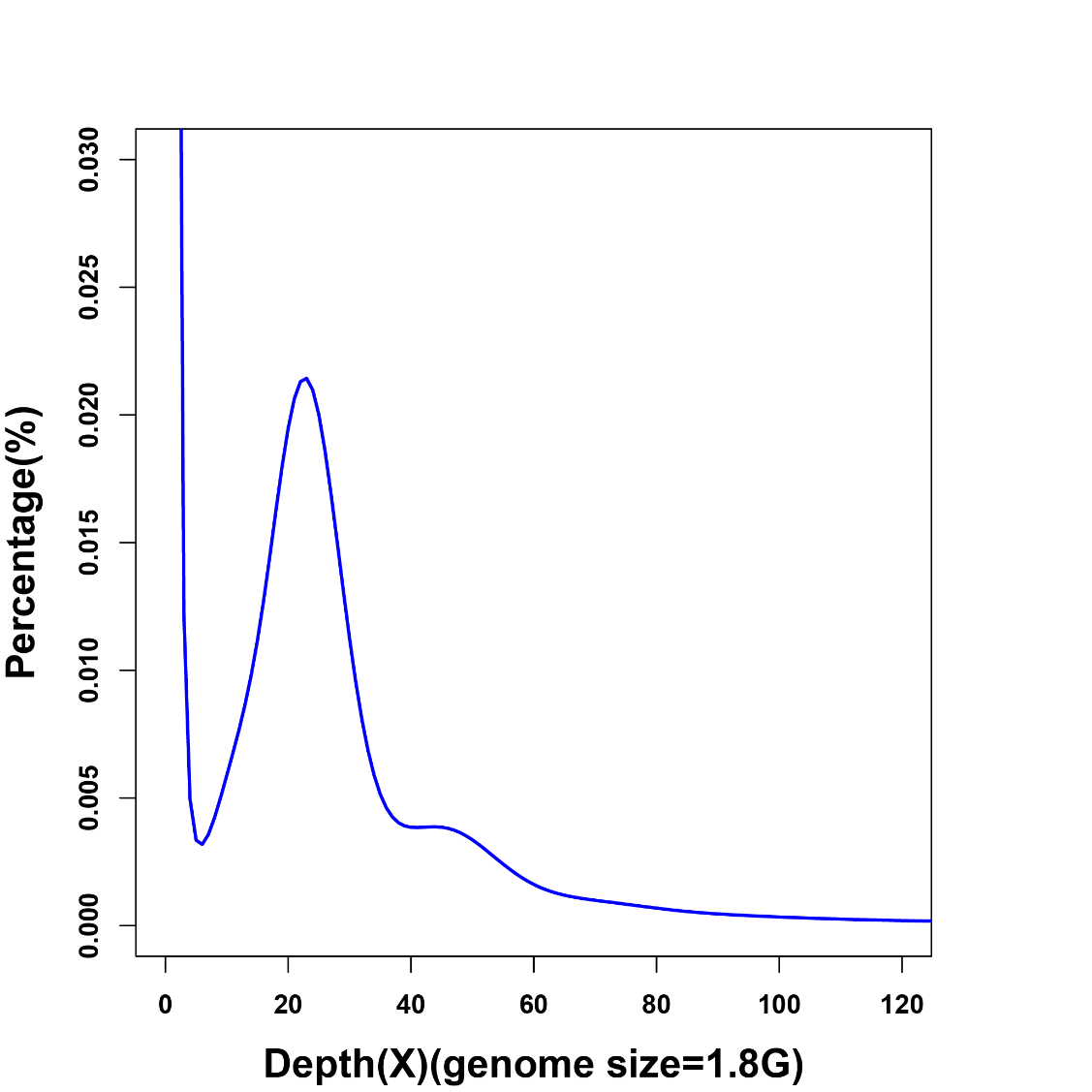


**Supplementary Figure 2** Genome survey of *S. maitianheensis* with two Illumina short-insert libraries (500 and 800 bp). The x-axis is the sequencing depth of each unique 17-mer, and the y-axis is the percentage of unique 17-mers. The k_num_ is 40,525,178,512 and the K_depth_ is 23.


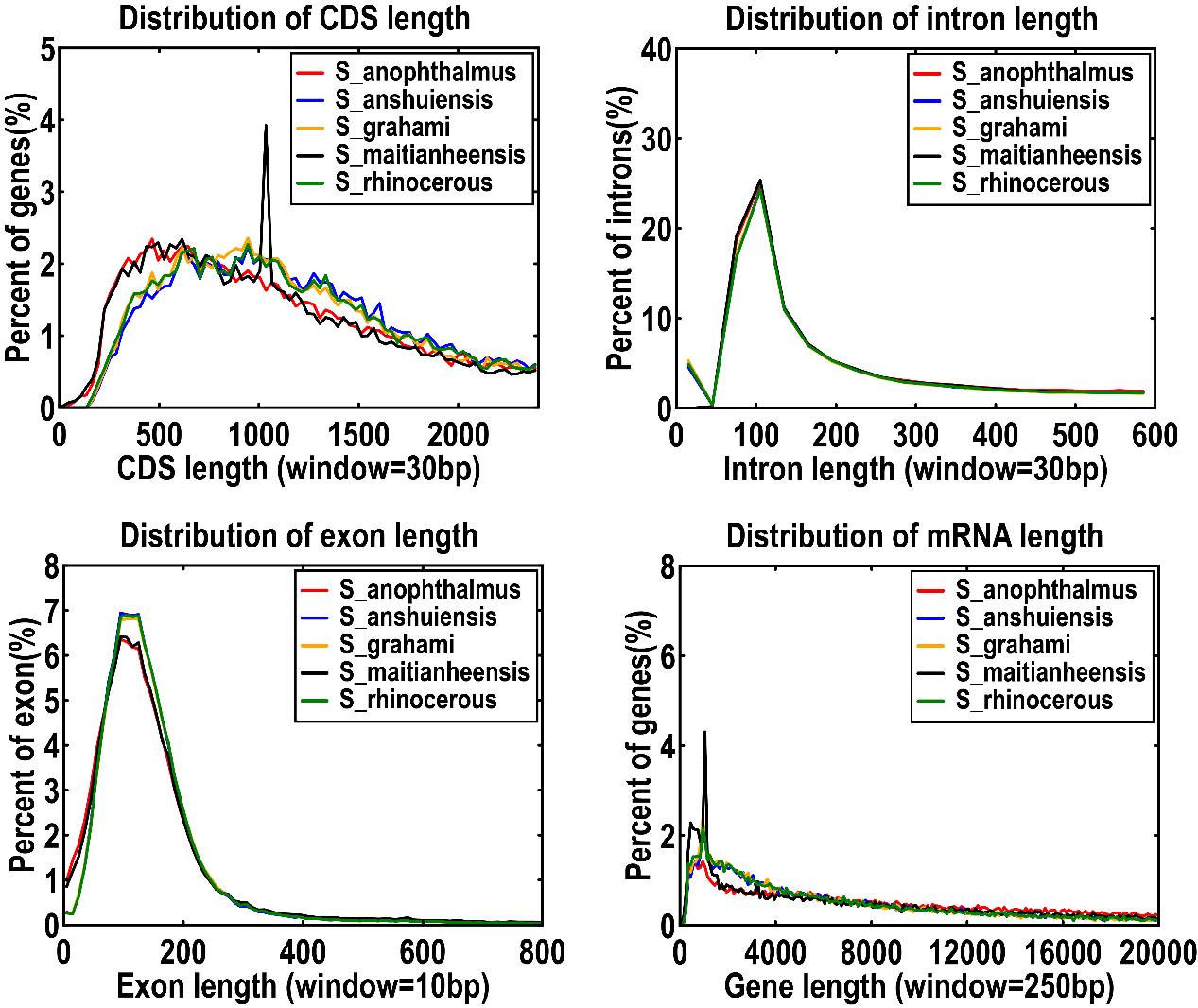


**Supplementary Figure 3** Comparisons of coding sequence (CDS), intron, exon and gene lengths of protein-coding genes in the five examined *Sinocyclocheilus* fishes.


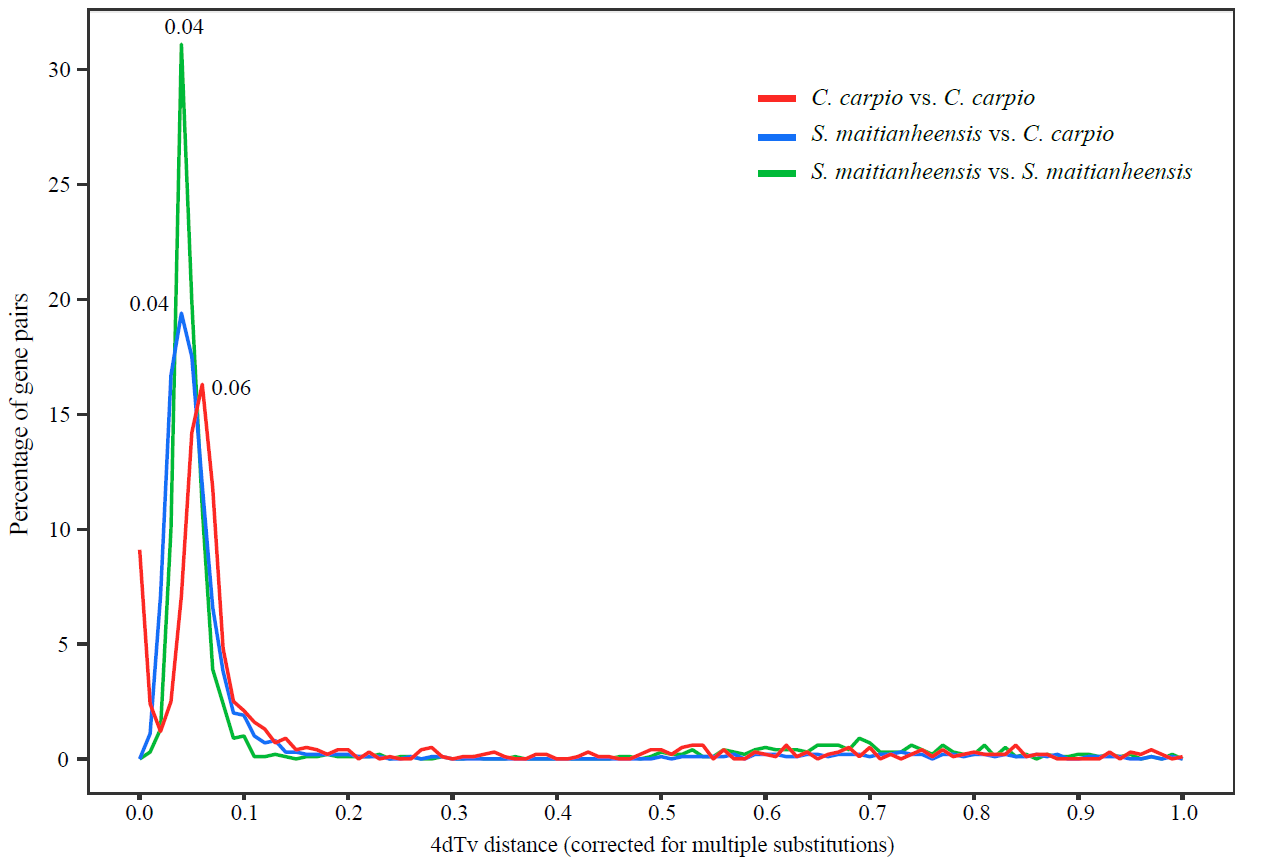


**Supplementary Figure 4** The 4dTv distributions of *C. carpio* vs *C. carpio*(red), *S. maitianheensis* vs *C. carpio* (bule), and *S. maitianheensis*vs *S. maitianheensis*(green). The WGD periods were calculated by using the following formula: [The recognized time of 3R WGD (~320 Mya) / The 4dTv peak values of 3R WGD in *Sinocyclocheilus* (0.65~0.75)] * The 4dTv peak values of lineage-specific WGD of*Sinocyclocheilus* (0.04~0.05).


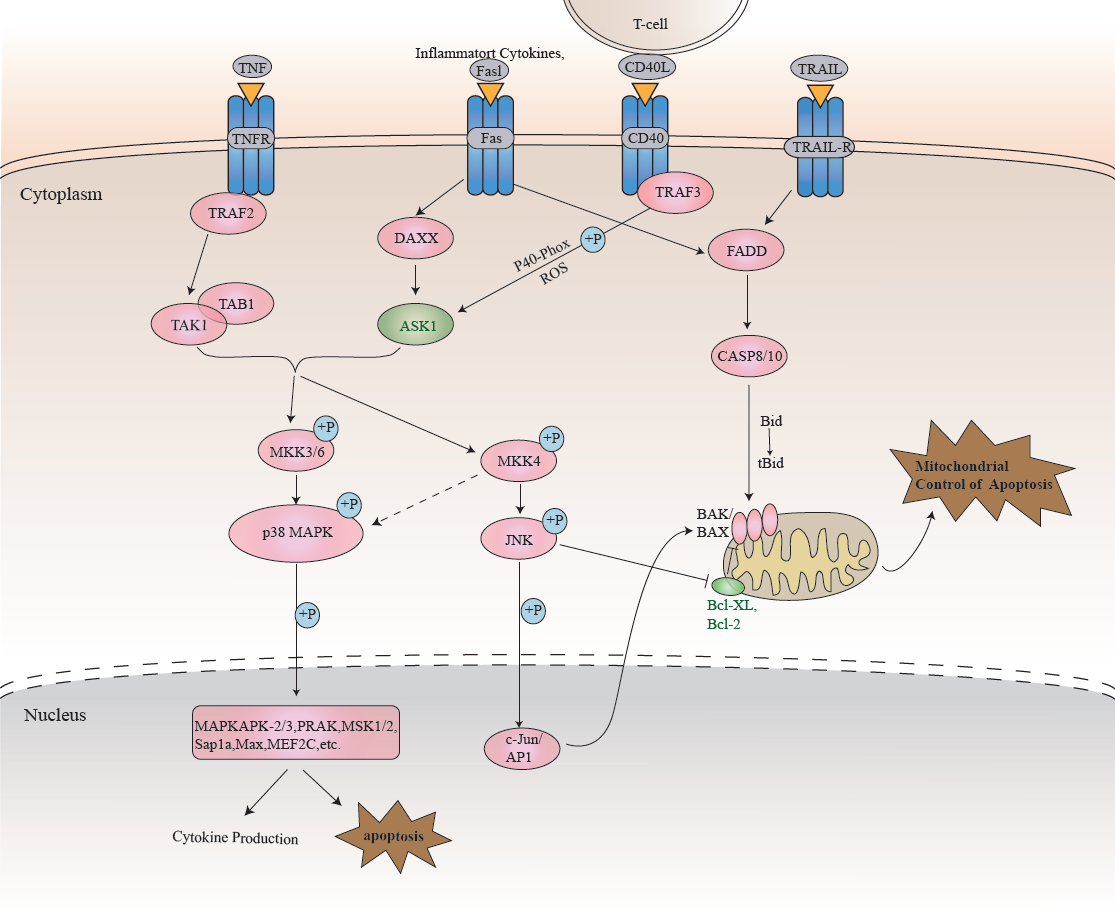


**Supplementary Figure 5** Proposed apoptosis caused by the p38 and mitochondrial pathways. Green color represents genes with reduced copy number in *S. anophthalmus*. Pink represents other genes in the two pathways. See full names of the abbreviated genes in the Abbreviations. Accession references include ko04210, ko04010, and Cell Signaling Technology (<https://www.cellsignal.com/pathways>).

**Supplementary Table 1** Accession IDs of 15 immune genes in the public Uniprot database.

| **Gene name** | **Accession ID** |
| --- | --- |
| *cd40* | B8X730 |
| *tnfa* | Q1L8A5 |
| *tak1* | A0A0R4IVJ5 |
| *tab1* | Q6NUU7 |
| *mkk4a* | Q9DGR7 |
| *mkk4b* | A4UU31 |
| *mkk6* | Q9DGE0 |
| *fas* | F1QG24 |
| *fasl* | Q0PKX7 |
| *fadd* | A0A0R4IXF0 |
| *daxx* | Q1LUU4 |
| *ask1* | E7F2L3 |
| *cd40l* | B8YI05 |
| *bcl2l1* | Q90Z98 |
| *bcl-2a* | Q564A4 |

**Supplementary Table 2** Summary of the assembled *S. maitianheensis* genome.

| **Parameter** | **Contig** | | **Scaffold** | |
| --- | --- | --- | --- | --- |
|  | **Size (bp)** | **Number** | **Size (bp)** | **Number** |
| N90 | 6,222 | 64,844 | 277,030 | 1,351 |
| N80 | 10,850 | 46,404 | 558,195 | 930 |
| N70 | 15,286 | 34,503 | 813,021 | 682 |
| N60 | 19,787 | 25,656 | 1,104,849 | 506 |
| N50 | 24,718 | 18,685 | 1,356,424 | 367 |
| Longest | 198,488 | | 12,546,551 | |
| Total Size | 1,542,228,659 | | 1,689,233,642 | |
| Total(>100bp) Number(>100bp) | 212,978 | | 106,737 | |
| Total(>2kb) Number(>2kb) | 93,111 | | 6,180 | |

**Supplementary Table 3** Comparisons among the genome assemblies of five *Sinocyclocheilus* fishes.

| **Species** | **Gnome size（Gb）** | **Contig N50 (bp)** | **Scaffold N50 (bp)** | **GC rate** | **Complete BUSCOs** |
| --- | --- | --- | --- | --- | --- |
| *S. maitianheensis* | 1.7 | 24,718 | 1,356,424 | 37.6% | 97.1% |
| *S. anophthalmus* | 1.9 | 229,819 | 309,865 | 38.6% | 94.4% |
| *S. grahami* | 1.8 | 29,335 | 1,155,972 | 37.5% | 93.4% |
| *S. anshuiensis* | 1.7 | 16,708 | 1,251,185 | 37.3% | 96.7% |
| *S. rhinocerous* | 1.7 | 17,658 | 894,603 | 37.2% | 95.8% |

**Supplementary Table 4** Statistics of repeat elements in *S. maitianheensis* genome*.*

| **Type** | **Repeat Size(bp)** | **% of Genome** |
| --- | --- | --- |
| ProteinMask | 68,255,106 | 4.0 |
| RepeatMasker | 284,838,973 | 16.9 |
| TRF | 59,390,162 | 3.5 |
| *De novo* | 586,074,331 | 34.7 |
| Total | 664,837,705 | 39.4 |

**Supplementary Table 5** Classification of repeat elementsin *S. maitianheensis*genome.

|  | **Repbase TEs** | | **TE Protiens** | | ***De novo*** | | **Combined TEs** | |
| --- | --- | --- | --- | --- | --- | --- | --- | --- |
| **Type** | **Length (bp)** | **% in genome** | **Length (bp)** | **% in genome** | **Length (bp)** | **% in genome** | **Length (bp)** | **% in genome** |
| DNA | 204,042,638 | 12.1 | 18,460,199 | 1.1 | 388,536,246 | 23.0 | 455,929,864 | 27.0 |
| LINE | 39,799,018 | 2.4 | 25,621,692 | 1.5 | 78,331,206 | 4.6 | 104,874,936 | 6.2 |
| SINE | 4,954,853 | 0.3 | 0 | 0 | 4,853,932 | 0.3 | 9,149,394 | 0.5 |
| LTR | 43,879,906 | 2.6 | 24,200,433 | 1.4 | 120,119,153 | 7.1 | 135,231,560 | 8.0 |
| Other | 9,331 | 0.0 | 0 | 0 | 0 | 0 | 9,331 | 0.0 |
| Unknown | 0 | 0 | 0 | 0 | 7,652,142 | 0.5 | 7,652,142 | 0.5 |
| Total | 284,838,973 | 16.9 | 68,255,106 | 4.0 | 557,203,090 | 33.0 | 614,259,898 | 36.4 |

**Supplementary Table 6** Protein-coding gene set of *S. maitianheensis*genome.

| **Gene Set** | | **Number** | **Average Transcript Length (bp)** | **Average CDS Length (bp)** | **Average Exons per Gene** | **Average Exon Length (bp)** | **Average Intron Length (bp)** |
| --- | --- | --- | --- | --- | --- | --- | --- |
| Homolog |  |  |  |  |  |  |  |
|  | *Danio rerio* | 37,977 | 11,198.0 | 1,293.0 | 6.9 | 187.0 | 1,675.0 |
|  | *Oryziaslatipes* | 68,761 | 7,479.0 | 1,060.0 | 4.7 | 224.5 | 1,726.0 |
| *Sinocyclocheilusanshuiensis* | | 73,170 | 8,825.0 | 1,241.0 | 5.9 | 212.1 | 1,562.0 |
| *Sinocyclocheilusrhinocerous* | | 43,007 | 7,270.0 | 1,024.0 | 4.5 | 226.7 | 1,777.0 |
| Transcriptome |  | 76,808 | 8,158.0 | 2,004.0 | 6.6 | 304.6 | 1,103.0 |
| MAKER | | 39,977 | 15,881.0 | 1,626.0 | 8.6 | 188.3 | 1,728.0 |
|  | |  |  |  |  |  |  |

**Supplementary Table 7** Function annotation of the *S. maitianheensis*gene set.

| **Parameter** | **Number** | **Percentage (%)** |
| --- | --- | --- |
| Total | 39,977 | 100% |
| InterPro | 32,538 | 81.4% |
| KEGG | 35,293 | 88.3% |
| Swissprot | 33,843 | 84.7% |
| TrEMBL | 36,668 | 91.7% |
| Overall | 38,677 | 96.8% |
| Unannotated | 1300 | 3.3% |

**Supplementary Table 8** BUSCO results of the protein-coding genein five *Sinocyclocheilus* fishes.

| **Species** | **Complete BUSCOs** | **Complete and single-copy BUSCOs** | **Complete and duplicated BUSCOs** | **Fragmented BUSCOs** | **Missing BUSCOs** |
| --- | --- | --- | --- | --- | --- |
| *S. maitianheensis* | 80.4% | 54.9% | 25.5% | 3.5% | 16.1% |
| *S. anophthalmus* | 85.9% | 48.8% | 37.1% | 4.9% | 9.2% |
| *S. grahami* | 94.6% | 49.9% | 44.7% | 2.9% | 2.5% |
| *S. anshuiensis* | 98.3% | 40.9% | 57.4% | 1.0% | 0.7% |
| *S. rhinocerous* | 97.3% | 39.9% | 57.4% | 1.5% | 1.2% |

**Supplementary Table 9** Statistics of orthogroupsin the ten representative species.

| Species | Genes number | Genes in orthogroups | Unclustered genes | Orthogroups number | Unique orthogroups | Average genes per orthogroup |
| --- | --- | --- | --- | --- | --- | --- |
| *S. grahami* | 42,109 | 38,746 | 3,363 | 16,453 | 42 | 2.4 |
| *S. maitianheensis* | 39,977 | 32,150 | 7,827 | 15,617 | 331 | 2.1 |
| *S. rhinocerous* | 42,377 | 37,476 | 4,901 | 17,128 | 124 | 2.2 |
| *S. anshuiensis* | 40,470 | 38,932 | 1,538 | 15,889 | 10 | 2.5 |
| *S. anophthalmus* | 49,865 | 38,969 | 10,896 | 17,414 | 304 | 2.2 |
| *Cyprinus carpio* | 49,264 | 37,661 | 11,603 | 16,907 | 433 | 2.2 |
| *Mus musculus* | 22,927 | 21,202 | 1,725 | 13,675 | 342 | 1.6 |
| *Oryziaslatipes* | 19,699 | 17,634 | 2,065 | 11,436 | 125 | 1.5 |
| *Homo sapiens* | 22,501 | 19,008 | 3,493 | 13,585 | 190 | 1.4 |
| *Scleropagesformosus* | 22,016 | 18,336 | 3,680 | 11,874 | 38 | 1.5 |
| *Danio rerio* | 26,458 | 25,215 | 1,243 | 14,750 | 118 | 1.7 |

**Supplementary Table 10** Copy numbers of 15 examined immune genes in10 representative vertebrate genomes.

| Species -gene | *tak1* | *tab1* | *ask1* | *fas* | *fasl* | *fadd* | *tnfa* | *daxx* | *mkk4a* | *mkk4b* | *mkk6* | *cd40* | *cd40l* | *bcl-2a* | *bcl2l1* |
| --- | --- | --- | --- | --- | --- | --- | --- | --- | --- | --- | --- | --- | --- | --- | --- |
| *S.anophthalmus* | 2 | 1 | 5(1)^*^ | 2 | 2 | 2 | 3 | 2 | 0 | 4 | 5 | 3 | 2 | 5 | 1 |
| *S.anshuiensis* | 2 | 1 | 6 | 2 | 2 | 2 | 2 | 2 | 2 | 2 | 4 | 2 | 2 | 4 | 2 |
| *S.grahami* | 2 | 1 | 6 | 2 | 2 | 2 | 2 | 2 | 1 | 4 | 4 | 3 | 2 | 4 | 2 |
| *S.maitianheensis* | 2 | 1 | 6 | 2 | 2 | 2 | 3 | 2 | 0 | 4 | 4 | 2 | 2 | 4 | 2 |
| *S.rhinocerous* | 2 | 1 | 6 | 2 | 2 | 2 | 3 | 2 | 3 | 3 | 3 | 2 | 2 | 4 | 2 |
| *C.carpio* | 2 | 2 | 7 | 2 | 2 | 2 | 2(1)^*^ | 2 | 1 | 5 | 4(1)^*^ | 2 | 2 | 4 | 2 |
| *D.rerio* | 1 | 1 | 3 | 1 | 1 | 1 | 1 | 1 | 1 | 2 | 2 | 1 | 1 | 2 | 1 |
| *O.latipes* | 1 | 1 | 3 | 0 | 0 | 0 | 0 | 1 | 1 | 2 | 1(1)^*^ | 0 | 0 | 1 | 1 |
| *S.formosus* | 1 | 1 | 3 | 0 | 0 | 0 | 0 | 1 | 2 | 1 | 4 | 0 | 0 | 2 | 2 |
| *A.mexicanus* | 1 | 1 | 3 | 0 | 0 | 1 | 0 | 1 | 2 | 1 | 1 | 0 | 0 | 1 | 1 |

* Predicted pseudogenes are provided in the brackets.

**Supplementary Table 11** Numbers of predicted AMP sequences.

| **Type of AMP-Species** | ***S. grahami*** | ***S. maitianheensis*** | | ***S. rhinocerous*** | | ***S. anshuiensis*** | ***S. anophthalmus*** |
| --- | --- | --- | --- | --- | --- | --- | --- |
| Thrombin | 136 | 95 | 119 | | 128 | | 116 |
| Histone | 61 | 36 | 69 | | 55 | | 75 |
| Lectin | 51 | 30 | 52 | | 50 | | 45 |
| Chemokine | 43 | 37 | 45 | | 39 | | 43 |
| Scolopendin | 39 | 33 | 46 | | 46 | | 36 |
| Ubiquitin | 35 | 23 | 34 | | 38 | | 47 |
| BPTI | 35 | 29 | 39 | | 31 | | 37 |
| Beta2-Microglobulin | 27 | 23 | 35 | | 19 | | 28 |
| Hemoglobin | 20 | 4 | 18 | | 21 | | 19 |
| Acipensin | 15 | 10 | 11 | | 9 | | 19 |
| Neuropeptide | 14 | 7 | 11 | | 14 | | 15 |
| Lysozyme | 9 | 5 | 8 | | 6 | | 7 |
| Antileukoprotease | 7 | 4 | 2 | | 4 | | 3 |
| Ribonuclease | 7 | 0 | 6 | | 4 | | 0 |
| Phospholipase_A2 | 6 | 3 | 6 | | 7 | | 4 |
| Synuclein | 5 | 1 | 4 | | 5 | | 5 |
| NK-lysin_fish | 4 | 7 | 6 | | 5 | | 4 |
| GAPDH | 4 | 2 | 3 | | 3 | | 3 |
| Skin-PYY | 4 | 3 | 4 | | 4 | | 4 |
| Amyloid | 4 | 2 | 4 | | 4 | | 3 |
| Ubiquicidin | 4 | 3 | 3 | | 4 | | 2 |
| Defensin | 3 | 2 | 8 | | 6 | | 6 |
| Peptide_3910_pig | 3 | 1 | 2 | | 2 | | 0 |
| Antiproteinase | 3 | 3 | 2 | | 3 | | 1 |
| Saposin-like | 2 | 2 | 2 | | 2 | | 4 |
| Amylin | 2 | 1 | 2 | | 2 | | 1 |
| sOT2_reptile | 2 | 0 | 3 | | 2 | | 2 |
| AJHbalpha_fish | 2 | 0 | 0 | | 1 | | 1 |
| LEAP-2 | 1 | 1 | 3 | | 2 | | 6 |
| Bothropstoxin | 1 | 0 | 0 | | 2 | | 1 |
| CcAMP1_insect | 1 | 1 | 0 | | 1 | | 1 |
| Ixodidin | 1 | 1 | 0 | | 1 | | 1 |
| hGlyrichin_syn | 1 | 1 | 2 | | 1 | | 0 |
| Thymosin | 0 | 3 | 2 | | 0 | | 5 |
| Waprin | 0 | 4 | 0 | | 0 | | 1 |
| Cerotoxin | 0 | 1 | 0 | | 0 | | 0 |
| cOT2_reptile | 0 | 0 | 0 | | 1 | | 0 |
| Hepcidin | 0 | 1 | 0 | | 0 | | 0 |
| total | 552 | 379 | 551 | | 522 | | 545 |
